# Cognitive Fitness: Harnessing the Strength of Exerkines for Aging and Metabolic Challenges

**DOI:** 10.3390/sports12020057

**Published:** 2024-02-13

**Authors:** Mona Saheli, Mandana Moshrefi, Masoumeh Baghalishahi, Amirhossein Mohkami, Yaser Firouzi, Katsuhiko Suzuki, Kayvan Khoramipour

**Affiliations:** 1 Department of Anatomical Sciences, Afzalipour Faculty of Medicine, Kerman University of Medical Sciences, Kerman 7616913555, Iran; saheli.mona@gmail.com (M.S.); fbaghalishahi@gmail.com (M.B.); 2 Department of Physiology and Pharmacology, Afzalipour Faculty of Medicine, Kerman University of Medical Sciences, Kerman 7616913555, Iran; mn.moshrefi@gmail.com; 3 Department of Exercise Physiology, Faculty of Sport Sciences, Hakim Sabzevari University, Sabzevar 9617976487, Iran; amirhosseinmohkami6@gmail.com; 4 Department of Exercise Physiology, Faculty of Sport Sciences, Shahid Bahonar University, Kerman 7616913439, Iran; yaserfiroz7@gmail.com; 5 Faculty of Sport Sciences, Waseda University, Tokorozawa 359-1192, Japan; 6 Neuroscience Research Center, Institute of Neuropharmacology, Kerman University of Medical Sciences, Kerman 7619813159, Iran

**Keywords:** exerkines, stem cells, cognition, elderlies, metabolic disorders

## Abstract

Addressing cognitive impairment (CI) represents a significant global challenge in health and social care. Evidence suggests that aging and metabolic disorders increase the risk of CI, yet promisingly, physical exercise has been identified as a potential ameliorative factor. Specifically, there is a growing understanding that exercise-induced cognitive improvement may be mediated by molecules known as exerkines. This review delves into the potential impact of aging and metabolic disorders on CI, elucidating the mechanisms through which various exerkines may bolster cognitive function in this context. Additionally, the discussion extends to the role of exerkines in facilitating stem cell mobilization, offering a potential avenue for improving cognitive impairment.

## 1. Introduction

Cognitive impairment, also known as CI, pertains to challenges or deficits in cognitive functions, which encompass various mental processes such as memory (i.e., the capacity to retain and retrieve information) [1], attention (i.e., the ability to concentrate on specific tasks or stimuli) [2], executive function (i.e., higher-order cognitive processes such as planning, organizing, decision-making, and problem-solving) [3], language (i.e., comprehension, expression, and communication through spoken and written language) [4], perception (i.e., interpreting and comprehending sensory information from the environment) [5], and spatial navigation (the ability to perceive and navigate the physical space surrounding us) [6]. CI can be classified into various degrees, spanning from normal cognitive function to mild, moderate, and severe impairment. This classification is frequently determined by diverse assessment tools and criteria, including the Mini-Mental State Examination (MMSE) and the American Geriatrics Society criteria. Here is a summary outlining the distinct degrees of CI:

Mild CI: Mild CI is characterized by a slight decline in cognitive abilities, which may not interfere with daily life. Individuals with mild CI may have difficulty with memory, language, or visuospatial skills, but they can still perform most daily tasks.

Moderate CI: Moderate CI is marked by a more significant decline in cognitive abilities, which can impact daily life and may require assistance in various tasks. Individuals with moderate CI may have difficulty with memory, language, visuospatial skills, and executive function.

Severe CI: Severe CI is characterized by a profound decline in cognitive abilities, which can render individuals unable to perform most daily tasks. This stage of CI may require more extensive support and assistance in various aspects of daily life [7,8,9,10].

The rate of cognitive disorders in different human societies varies due to several factors, including genetic factors, ethnicity, geographical location, physical activity, gender, nutrition, and suffering from metabolic diseases. These factors collectively play a role in the prevalence of cognitive impairment. Some of these factors have a direct impact on cognitive function, while others influence the prevalence of cognitive disorders through their effects on health and lifestyle.

1. Genetic factors: genetic defects play a significant role in the development of cognitive disorders, with some genetic factors increasing the risk of cognitive impairment and dementia.
2. Ethnicity: Different ethnic groups may have varying susceptibility to cognitive disorders due to genetic factors and cultural influences. For example, individuals of African descent are at a higher risk of developing Alzheimer’s disease (AD)‚ while individuals of Asian descent may have a lower risk [11].
3. Geographical location: The prevalence of cognitive disorders can differ across regions due to factors such as access to healthcare, education, and lifestyle. For instance, cognitive disorders may be more common in urban areas with higher levels of pollution and stress [12].
4. Physical activity: Engaging in regular physical activity has been shown to reduce the risk of CI and improve cognitive function. Studies have found that engaging in light physical activity (LPA) for at least 3 h per day can reduce the chance of CI in older adults [13,14].
5. Gender: gender has been found to play a role in the prevalence of cognitive disorders, with some studies suggesting that women are at a higher risk of developing AD [15].

A study conducted in Hawaii found that a higher proportion of women than men had AD, with the highest female prevalence among Native Hawaiians and Pacific Islanders (NHPI) [14]. Another study found that 60.8% of women over 64 years old had cognitive deficits, while 36% of men in the same age group had cognitive deficits. However, it is essential to note that these percentages are based on a small sample size and may not be representative of the general population.

6. Nutrition: Dietary factors play a crucial role in cognitive function and prevention of cognitive disorders. A diet rich in anti-inflammatory, low-sugar, and minimally processed foods, as well as a proper omega-6 to omega-3 ratio, can help maintain cognitive health [11].
7. Metabolic diseases: suffering from metabolic diseases, such as diabetes and obesity, has been associated with an increased risk of CI and dementia [12].

### 1.1. Aging, Metabolic Disorders, and CI

The pathophysiological link between aging and CI encompasses intricate fundamental interactions and extends to include the correlation between metabolic disorders and CI, as discussed below [16,17,18].

#### 1.1.1. Brain Atrophy

The process of normal aging encompasses a diverse range of changes, both functional and structural, within the brain, accompanied by declines across various cognitive domains [19,20]. The “disconnection hypothesis” seeks to elucidate the relationship between these age-associated alterations in cognition and brain structure. This hypothesis posits that disruptions in the communication pathways between different cortical regions can contribute to the observed cognitive decline [20,21]. A key factor potentially underlying this disconnection is the integrity of white matter (WM) in the brain. Existing evidence already indicates a correlation between compromised WM integrity and cognitive decline during the course of normal aging. This decline in WM integrity can be attributed to specific neural morphological alterations [22,23] that occur with aging [24]. Such morphological changes encompass a reduction in dendritic length and branching, decreased density of spines and synapses, and shifts in the distribution of spine subtypes [25,26,27,28]. These alterations are not merely structural; they have functional implications. Specifically, the loss or modification of dendritic spines and changes in their subtype distribution [29] can influence excitatory synaptic activity within neuronal networks. Such alterations in synaptic function within these networks are critical for maintaining optimal cognitive processing capabilities in normal aging individuals [25,26].

#### 1.1.2. Neuroplasticity

Neuroplasticity, contingent upon activity, represents a pivotal characteristic of the nervous system, affording neurons the capacity to communicate and adjust connections based on prior experiences. Synaptic plasticity, in general, adheres to distinct developmental and aging trajectories in individuals without underlying health complications. However, disruptions in plasticity are implicated in various neuropsychiatric disorders, including CI. Mechanistically, synaptic plasticity operates across diverse spatial and temporal scales, spanning from microseconds to a lifetime and from microscopic synapses to the entire nervous system [30]. 

Physical exercise has emerged as a robust modulator of neuroplasticity, demonstrating a significant impact on the growth of new connections between cells in crucial cortical areas of the brain. This phenomenon, intricately linked to brain plasticity, extends its benefits to both healthy and diseased states. The mechanisms underlying exercise-induced neuroplasticity involve the elevation of trophic factors, modulation of cerebrovascular function, and the reduction of toxic Aβ and tau proteins, collectively contributing to the optimization of cognitive function and neuronal resilience [31]. However, we still need more studies to understand exactly how different types of exercise affect neuroplasticity.

#### 1.1.3. Neurogenesis

Throughout a person’s lifetime‚ the hippocampus produces fresh neurons using neural stem cells (NSCs). These newly formed neurons have a crucial role in regulating mood and cognitive flexibility. Nevertheless, as one gets older, the generation of new neurons in the hippocampus decreases substantially due to age-related issues affecting NSC function. The aging brain experiences diminished neurogenesis primarily due to the significant decline in NSC activity, marked by enhanced quiescence and diminished proliferation of NSC.

#### 1.1.4. Neurotransmitter Changes

An imbalance in various neurotransmitter pathways can contribute to CI. This imbalance involves disruptions in key neurotransmitter systems such as the monoaminergic, GABAergic, histaminergic, and cholinergic systems, each characterized by specific neurotransmitters (e.g., acetylcholine and dopamine) [31,32,33] and receptors that regulate cognitive functions [33].

In addition, the intricate balance of neurotransmitter receptors within the brain is subject to a dynamic regulatory framework [34,35]. Factors such as receptor activation, gene expression patterns, and external stimuli play pivotal roles in governing the production and degradation of these receptors [36].

#### 1.1.5. Amyloid Plaques (Aβ) and Tau Tangles

Unusual clusters of proteins, known as amyloid plaques (made up of the Aβ protein) and tau tangles (resulting from the excessive phosphorylation of tau protein), tend to build up in the brains of those affected by CI [37]. It is worth noting that these protein aggregates can be detected in the hippocampus and cerebral cortex of certain elderlies [38] and individuals with metabolic disorders [39]. These clusters have been linked to neural harm and the deterioration of cognitive abilities [40].

Tau is a vital protein that is closely associated with microtubules and is typically found in neurons. Its main role is to maintain the stability of microtubules when everything is functioning normally. However, in pathological situations, tau tends to separate from microtubules and becomes involved in various neurological disorders, which are known for causing irreversible damage to neurons. These disorders include necrosis, apoptosis, necroptosis, pyroptosis, ferroptosis, autophagy-dependent neuronal death, and phagocytosis by microglia.

Researches indicate that the buildup of Aβ has been connected to difficulties in episodic memory, executive function, processing speed, visuospatial function, and overall cognition. The accumulation of Aβ and tau proteins can occur either independently or simultaneously in conditions such as primary age-related tauopathy and AD. Despite numerous efforts, our comprehension of the reciprocal influences of Aβ and tau accumulations on clinical characteristics remains incomplete [41].

#### 1.1.6. Oxidative Stress and Inflammation

Cellular damage and CI can result from these mechanisms, which also play a role in neurodegeneration. CI is heavily influenced by oxidative stress, where the generation of reactive oxygen species (ROS) surpasses their removal. This imbalance is greatly responsible for chronic inflammation. In elderlies [42] and individuals with metabolic disorders [43], mitochondrial dysfunction occurs alongside reduced activity of antioxidant enzymes, leading to heightened ROS levels. ROS is responsible for harmful effects on lipids, proteins, and nucleic acids, including DNA. This damage also takes place in the mitochondria, the primary source of ROS. As a result, a detrimental cycle is initiated, resulting in depleted energy and eventual cell demise. This phenomenon is observed in both peripheral tissues and the brain [42]. Moreover, the augmented generation of ROS results in the impairment of the blood-brain barrier (BBB) function and the escalation of BBB permeability. Consequently, the defense mechanism against external toxins, pathogens, and pro-inflammatory cytokines becomes compromised, triggering an intensified activation of microglia, neuroinflammation, deposition of amyloid, and infliction of damage upon neurons [44].

Peripheral pro-inflammatory cytokines like IL-1 and TNF-α, along with macrophages, have the ability to breach the BBB and stimulate the brain’s microglia—macrophages that reside in the brain. This activation results in neuroinflammation, worsens amyloid buildup, and causes harm to neurons [45]. Additionally, inflammation throughout the body harms the BBB and causes it to become more permeable, diminishing its ability to shield against harmful external substances like toxins, pathogens, and pro-inflammatory cytokines [46]. The initiation of CI relies on the dysfunctional BBB, which plays a crucial role in clearing amyloid from the brain and transferring it into the bloodstream. The accumulation of amyloid in the brain, perceived as a significant pathology, can be attributed to the malfunctioning BBB [47].

#### 1.1.7. Vascular Changes and Dysfunction

The brain’s blood flow can be hindered by age-related alterations in blood vessels, as well as metabolic disorders, causing a scarcity of oxygen and nutrients for brain cells. This can lead to CI and a higher susceptibility to medical conditions such as vascular dementia. Vascular aging encompasses a range of transformations in blood vessels as a person ages, including heightened rigidity, modifications in vessel walls, diminished ability for blood vessel growth, and impaired capability for endothelium-driven vasodilation. Individuals who are at risk for or already have cardiovascular disease may experience these changes associated with aging earlier than others [48]. These changes are known as early or premature vascular aging and are primarily caused by damage to the large and small vessels in the brain. This type of aging in the blood vessels is a significant contributor to CI that is commonly observed with increasing age [49].

#### 1.1.8. Mitochondrial Dysfunction 

Aging and metabolic disorders deteriorate mitochondrial function, resulting in a diminished ability to supply sufficient energy to brain cells. This decline in mitochondrial function could potentially play a key role in the development of CI [50]. The mitochondrial cascade hypothesis suggests that the pathogenesis of cognitive decline is closely linked to mitochondrial dysfunction. This is because the initial state of mitochondrial function and the rate at which it changes over time both have a significant impact on the progression of cognitive decline [51].

#### 1.1.9. Insulin Resistance

Insulin resistance, as indicated by various studies, has been linked to both CI and type 2 diabetes. The brain heavily relies on insulin for sustaining memory and cognitive abilities at optimal levels. Any interference caused by insulin resistance may potentially disrupt these vital functions, ultimately paving the way for the onset of CI [52].

#### 1.1.10. Brain Energy Metabolism

Memory and other cognitive processes may be affected when metabolic disorders interfere with the brain’s energy metabolism [43].

#### 1.1.11. Advanced Glycation End Products (AGEs)

Elevated blood sugar levels have the potential to give rise to advanced glycation end products, which have the ability to amass in the brain and result in oxidative stress and inflammation. These factors are known to play a role in CI [53].

### 1.2. Management of CI

There have been multiple studies exploring various ways to combat CI. A systematic review and meta-analysis found no consistent evidence of a benefit for any pharmacologic agent in preventing and treating CI in older adults and individuals with metabolic disorders. As a result, non-pharmacological approaches are considered desirable for preventing and treating CI. One of the standout methods is engaging in physical activity and exercise, which has been touted as a secure, successful, and productive strategy that not only addresses but also prevents CI [54].

Fitness levels have been shown to have a significant impact on brain structure and function, particularly in older adults. Adequate physical activity and maintaining aerobic fitness are critical for cognitive function and brain health, especially during preadolescence, when the brain is undergoing significant changes in structure and function related to cognition, including executive control and relational memory [24,55,56,57,58]. Higher cardiorespiratory fitness levels are associated with greater gray matter volume in the prefrontal cortex and hippocampus in older adults. Also, it has been positively associated with white matter microstructure, particularly in frontal areas of the brain. Consistent positive findings suggest a promising role for physical activity in promoting hippocampal structure and function throughout the lifespan. Studies showed that cardiorespiratory fitness is positively associated with brain network integrity and measures of network integration and specialization in older adults. Cardiorespiratory fitness has also been associated with cognitive function across multiple domains, including memory, in older adults [24,56,57,59].

Physical exercise has been associated with increased neuroplasticity, neurotrophic factors, and improvements in brain function. It promotes the growth of new connections between cells in important cortical areas of the brain, which is linked to brain plasticity [36].

It benefits neuroplasticity in both health and disease stages by targeting different aspects, such as increasing trophic factors, changing cerebrovascular function, and lowering toxic Aβ and tau proteins [34].

Despite numerous studies indicating exerkines as the primary factor, the detailed mechanisms behind this phenomenon remain incompletely elucidated. Our objective was to furnish compelling evidence showcasing the beneficial impact of physical exercise on cognitive improvement (CI).

## 2. Exerkines and CI

The advantageous consequences of physical activity could potentially be attributed to the role of exerkines [60]. Exerkines encompass signaling molecules that are generated as a result of short-term and/or long-term exercise, operating through endocrine, paracrine, and/or autocrine routes to elicit their influences. Various factors are released by a diverse range of organs, cells, and tissues. These factors include myokines from skeletal muscle, cardiokines from the heart, hepatokines from the liver, adipokines from white adipose tissue, baptokines from brown adipose tissue, and neurokines from neurons. The subsequent section will aim to explore the potential impact of exerkines on enhancing CI (Table 1) [61].

### 2.1. IL-6

Initially, interleukin-6 (IL-6) was acknowledged for its notable anti-inflammatory function. However, IL-6 is a pleiotropic factor produced by various cell types, targeting numerous cell types and exerting a wide range of opposing biological effects. It possesses the ability to induce several distinct intracellular signaling pathways [62,63,64,65,66]. Exercise has sparked the idea that IL-6, which is released by skeletal muscle, may play a part in the metabolic changes and endurance enhancements brought on by physical activity. In fact, IL-6 has been designated as an energy detector, aiding in the regulation of blood sugar levels by promoting the breakdown of glucose and fatty acids. Additionally, the rise of IL-6 stimulates the growth of new neurons in the hippocampus [67,68]. All of these effects are believed to be induced when IL-6 is secreted by muscles during exercise, exhibiting anti-inflammatory properties [69].

### 2.2. Cathepsin B

The exercise-induced muscle secretory factor known as Cathepsin B (CTSB) is a cysteine protease that plays a role in hippocampal functions. In mice, CTSB is able to cross the blood-brain barrier (BBB) and is responsible for a significant increase in brain-derived neurotrophic factor (BDNF) and doublecortin (DCX) in the central nervous system. The neuroprotective effects of both proteins are closely tied to hippocampal neurogenesis and neuronal migration. Additionally, CTSB plays a role in mitochondrial cell death signaling by controlling the release of proapoptotic molecules [70].

Lysosomes are pivotal in the breakdown of certain detrimental proteins like Aβ and in upholding the equilibrium of intracellular protein. The malfunctioning of lysosomes has been perceived as a key flaw in neurons, resulting in the advancement of CI [36]. Prolonged physical activity raised the levels of fully developed cathepsin L/D enzymes within the cortex of mice with AD‚ indicating that exercise successfully rehabilitated the impaired lysosomal function in CI and reinstated the flow of autophagy [70]. AD leads to a decline in the levels of mature Cathepsin L and Cathepsin D, whereas the immature variants exhibit atypical surges. This suggests that the impairment of lysosomal hydrolase maturation occurs in AD. The development of maturity in lysosomal hydrolases is reliant on the transportation of vesicles to the lysosome and the subsequent process of cleavage. AD has been associated with impaired vesicular trafficking, according to prior research [71].

The relationship between exercise, CTSB, and cognitive function has been the subject of several studies. These studies concluded that aerobic exercise is more effective than resistance exercise in elevating CTSB levels and improving cognitive function [55,56,72,73,74].

### 2.3. Brain-Derived Neurotrophic Factor (BDNF)

BDNF, released by both the brain and muscles, belongs to the neurotrophin group, which signifies its vital function in neuronal differentiation and survival, synaptic integrity, brain plasticity, and memory. BDNF is crucial for the growth, preservation, and persistence of neurons, as well as for the adult production of new nerve cells in the hippocampus’ dentate gyrus [75]. BDNF, in addition to promoting long-term potentiation—an essential process for learning and memory—has also been found to enhance the creation of new mitochondria within hippocampal neurons [76]. It is worth mentioning that BDNF demonstrates its ability to boost mitochondrial biogenesis in these specific brain cells [70,77].

Older adults who engaged in aerobic exercise for one year, specifically 40 min of walking at 60–75% maximum heart rate reserve‚ three times a week‚ experienced a noteworthy 2% increase in the volume of their hippocampus. This increase is directly associated with improvements in aerobic fitness, as indicated by the correlation coefficient of 0.37 and 0.40 for the left and right hippocampus, respectively, in relation to VO_2_ max. The levels of BDNF in the serum show a correlation coefficient of 0.36 and 0.37 for the left and right hippocampus, respectively. Similarly, the correlation coefficient for performance on a spatial memory task is 0.23 and 0.29 for the left and right hippocampus, respectively [78]. Given that the hippocampus tends to diminish by 1–2% each year in elderly individuals without dementia [79], it can be inferred from the aforementioned findings that engaging in aerobic exercise for a year might potentially counteract the shrinkage of the hippocampus associated with aging by a period of 1–2 years [78].

A study reported that BDNF response to exercise is intensity-dependent, with intense exercise being the most effective. In a study on young college students, HIIT improved cognitive function to a higher degree compared to moderate-intensity continuous training (MICT), with cognitive improvements correlating with lactate release. However, a study on people with spinal cord injury (SCI) found that acute submaximal exercise did not impact plasma or serum BDNF levels or cognitive function, suggesting that the relationship between exercise intensity and BDNF in individuals with CI may be more complex [80,81,82].

### 2.4. Irisin

Irisin, derived from the cleavage of fibronectin type III domain-containing protein 5 (FNDC5), is a transmembrane glycoprotein with 112 amino acids. It is aptly named after Iris, the Greek goddess of messengers [83]. Following the proteolytic cleavage process separating it from FNDC5, irisin is released and operates as a myokine [84]. Although irisin was primarily discovered in skeletal muscles, subsequent findings revealed its expression in several tissues, encompassing the brain [85]. Exercise increases the production of a transcription factor called PGC-1α in muscles. This, in turn, enhances the expression of a protein called FNDC5, leading to the release of more irisin into the bloodstream through the cleavage of FNDC5. The level of irisin in the plasma is elevated in individuals who engage in aerobic training [86]. Specifically, exercise stimulates the production of FNDC5 and BDNF in the hippocampus. This mechanism is dependent on PPARγ coactivator 1α (PGC-1α). Recent studies have shown that FNDC5/irisin activates the cyclic adenosine monophosphate/protein kinase B/cAMP response element-binding protein (cAMP/PKA/CREB) pathway in both mice and human brain tissue. Importantly, the stimulation of PGC-1α by FNDC5/irisin highlights the role of irisin as a crucial molecule facilitating communication between muscles and the brain [87]. The increase in FNDC5/irisin levels is accompanied by the promotion of hippocampal neurogenesis. Exercise-induced elevation of irisin leads to cell proliferation and BDNF expression in brain tissue, contributing to the generation of new neurons [68].

Furthermore, irisin exhibits anti-inflammatory properties by reducing the secretion of cytokines IL-6 and IL-1β from astrocytes cultured in the laboratory. Astrocytes that have been exposed to irisin provide protection to neurons against the toxic effects of Aβ [88].

Recent evidence supports the potential of exercise, particularly high-intensity training, to positively impact cognitive function in individuals with CI [89,90].

### 2.5. Apelin

Apelin, known as the naturally occurring ligand for the APJ orphan G protein-coupled receptor, was initially discovered in 1998. Both apelin and APJ are found throughout the body and serve as crucial factors in safeguarding cells within various organs. Apelin’s significance extends to conditions like cardiovascular disease, obesity, and cancer. Furthermore, apelins are present throughout the nervous system and have been identified to possess neuroprotective properties and have a positive effect on CI [88]. A study involving eleven obese non-diabetic males demonstrated that an eight-week endurance training program resulted in an increase in apelin expression in the muscles [91].

### 2.6. Clusterin

Clusterin, alternatively called apoJ, is a versatile protein that functions as a natural Chaperone. It displays involvement in numerous physiological and pathological conditions, including CI. The therapeutic impact of clusterin on patients with CI arises from its ability to alter Aβ aggregation and neuroinflammation. Notably, clusterin potentially holds a significant influence in facilitating Aβ clearance across the BBB [68,92]. Engaging in a disciplined workout routine for half a year (voluntary exercise) resulted in raised levels of plasma clusterin. Interestingly, these increased levels were closely related to enhancements in stamina and aerobic ability. The elevated clusterin levels were found to play a role in suppressing the interferon and cytokine signaling pathways within brain endothelial cells, consequently mitigating both chronic and immediate neuroinflammation [93,94].

Studies found that exercise plasma collected from running mice and infused into sedentary mice reduces baseline neuroinflammatory gene expression and experimentally induced brain inflammation and that plasma proteomic analysis revealed a concerted increase in complement cascade inhibitors, including clusterin [72]. Additionally, a study found that aerobic exercise increases plasma clusterin, and there is a positive association between changes in plasma clusterin and cardiorespiratory fitness as a response to aerobic exercise in older adults with AD [95]. These findings suggest clusterin could mediate the positive effect of exercise on CI [72,90].

### 2.7. C-X3-C Motif Chemokine Ligand 1 (CX3CL1)

A myokine commonly referred to as CX3CL1 was discovered in previous studies [96]. Recent research has shown that mRNA levels of CX3CL1 were elevated in muscles, while its protein levels were found to be increased in plasma following both intense endurance and resistance workouts [84,97]. CX3CL1, as indicated by these discoveries, is a myokine triggered by physical activity, potentially facilitating inter-organ communication between skeletal muscles and various bodily systems. In the realm of the brain, CX3CL1 could potentially inhibit neuroinflammation by activating CX3CR1, a receptor found in microglial cells specific to the C-X3-C motif chemokine [88,98].

While the specific effect of exercise intensity on CX3CL1 in the context of CI is not directly addressed in the available literature [99], the studies provide insights into the potential impact of exercise and related peptides on neuroinflammation and immune responses [90,95,99], which are relevant to the broader understanding of the topic. Further research specifically focusing on the relationship between exercise intensity, CX3CL1, and CI is warranted to draw more definitive conclusions.

### 2.8. FGF2 

FGF2, also called bFGF and FGF-β, is a crucial growth factor involved in the growth and multiplication of neural stem cells and their progenitors, promoting neural development. Its expression is widespread in different tissues, like the brain and muscles. Notably, engaging in aerobic exercise is shown to elevate FGF2 expression in animal models. FGF2, a neurotrophic factor, has the ability to promote the growth of new neurons and blood vessels in the brains of both adults and developing individuals [88,100].

### 2.9. FGF21

The hormone FGF21, a member of the FGF superfamily, was detected in mouse embryos in 2000. Its primary expression occurs in the liver, but it is also generated in different organs like muscle, adipose tissue, pancreas, and heart. FGF21 plays a pivotal role in maintaining energy balance through autocrine, paracrine, or endocrine mechanisms [101,102]. Preclinical research has shed light on the potential therapeutic application of FGF21 in managing metabolic disorders like diabetes. This is attributed to its ability to enhance insulin sensitivity, ameliorate glucose tolerance, and facilitate weight loss [103]. One notable consequence of exercise is the rise in FGF21 levels in the bloodstream, primarily driven by augmented FGF21 production in the liver. FGF21 has exhibited impressive protective qualities in numerous brain damage studies. It effectively counteracted inflammation and maintained the integrity of the blood-brain barrier by stimulating PPAR-γ activation. Additionally, it facilitated the growth of new blood vessels, effectively promoting neovascularization [104,105]. Furthermore, FGF21 has demonstrated its neuroprotective abilities, displaying anti-inflammatory and antioxidant properties. It significantly hindered the formation of amyloid plaques, neurofibrillary tangles, and overall neurodegeneration [105,106,107].

### 2.10. IGF-1

Exercise-induced neurogenesis is dependent on the presence of insulin-like growth factor 1 (IGF-1), which is also known as somatomedin C. This secreted peptide, structurally resembling insulin, plays a vital role in a range of physiological processes. It was further shown through a study that IGF-1 is crucial for facilitating exercise-induced neurogenesis [88].

Exercise has been shown to have a positive impact on cognitive function and IGF-1 levels. In a study conducted on female Wistar rats, resistance exercise training ameliorated MCI, and this improvement was attributed to the exercise-induced enhancement of IGF-1 protein and GST activity in the brain [108].

A systematic review of experimental studies in the elderly investigated the effects of exercise on IGF-1 levels and cognition. The review found disparities in the impact of exercise on IGF-1 levels, with some studies showing an increase, some showing no effect, and one showing a reduction in IGF-1 levels [109]. The review concluded that the type of physical exercise, protocols, and sample characteristics may explain these discrepancies. A study investigated the effect of resistance exercise intensity on the expression of IGF-1 in human skeletal muscle. The results showed that IGF-1 expression significantly increased following a higher intensity resistance exercise session compared to a lower intensity session. This suggests that the intensity of resistance exercise can impact the expression of IGF-1 in skeletal muscle [72].

### 2.11. LIF

Leukemia-inhibitory factor (LIF), a cytokine belonging to the interleukin-6 family, possesses versatile functions and was initially recognized for its ability to prompt macrophage differentiation. LIF holds significance in facilitating the growth, development, and viability of diverse cell varieties, which encompass neurons, myoblasts, hepatocytes, adipocytes, megakaryocyte progenitors, and myeloid cells. LIF has received acknowledgment as a myokine, demonstrating an escalated expression in reaction to exercise in both human and animal subjects [110,111]. The release of this entity from cells is regulated by a signaling peptide [112], and its ability to be secreted has been verified in experiments with human myotube cultures and mouse skeletal muscles [113]. A study by Besse-Patin et al. revealed that after a 3-h aerobic exercise session, there was up to four-fold increase in the expression of LIF mRNA in muscles, followed by a gradual decrease. A recent study discovered that static exercise led to a rise of nearly 50% in the plasma concentration of LIF, whereas dynamic exercise did not show any such increase. This indicates that the regulation of LIF expression may differ based on the type of exercise performed [114]. In addition, the application of ionomycin, an ionophore that transports Ca2 ions, resulted in increased levels of LIF mRNA and protein expression in muscle cells of humans [110]. The suggestion is that variations in Ca2+ levels after muscle contraction might have an impact on the transcription of LIF. Moreover, the PI3K-Akt pathway was found to influence LIF in human myotubes in culture, while the levels of JunB and c-Myc, which are stimulated by LIF, increased in skeletal muscles after resistance exercise [115].

### 2.12. VEGF 

Adult neurogenesis in the dentate gyrus of the hippocampus is bolstered by vascular endothelial growth factor (VEGF), which works in part by stimulating angiogenesis, the process of generating new blood vessels. This is important because neurogenesis takes place in an environment conducive to angiogenesis, ensuring an adequate supply of oxygen and nutrients [77].

According to research, engaging in wheel running was discovered to enhance the creation of new neurons in the hippocampus in adult mice by amplifying the presence of VEGF in their blood [116]. In a separate study, it was revealed that 12 weeks of aerobic exercise in human participants—encompassing activities such as ergometer cycling‚ treadmill running, stair climbing, and The surge in cerebral blood volume (CBV) within the dentate gyrus showed a significant correlation (correlation coefficient = 0.662) with this improvement. Consequently, the boosted CBV led to improved results in a short-term memory assessment (correlation coefficient = 0.62) [117].

One study found that high-altitude exposure (3540 m asl) of Long Evans rats during early adulthood impaired spatial and visual memory but prevented CI when the rats were housed in an enriched environment with a running wheel for voluntary exercise. The study suggests that exercise can prevent CI via VEGF signaling [118].

Another study evaluated the relationship between VEGF and cognitive improvements following exercised-primed transcranial direct current stimulation in MCI and AD [48,118].

### 2.13. Glial-Cell-Line-Derived Neurotrophic Factor (GDNF)

GDNF, another neurotrophic factor, has been demonstrated to rescue neurons from naturally occurring cell death as well as axotomy-induced cell death. Exercise has been associated with elevated GDNF protein concentrations in skeletal muscle, which can then be released into the bloodstream, cross BBB, and reach CNS. Studies have revealed a dose-response pattern for GDNF in response to exercise [119]. For instance, low-intensity exercise resulted in an increase in GDNF in the soleus, primarily a slow-twitch muscle, while decreasing GDNF content in the extensor digitorum longus (EDL), predominantly a fast-twitch muscle. Increasing the intensity of exercise, such as using running wheels with added resistance, led to clear evidence of recruitment (e.g., hypertrophy) in fast-twitch muscles and an associated increase in GDNF content [120].

### 2.14. 3-Hydroxybutyrate (3OHB)

Exerkines are not exclusive to proteins. Other types of molecules that participate in regulating physiological functions are also stimulated by vigorous exercise. Following the activation and breakdown of fatty acids, the liver generates ketone bodies, particularly 3-hydroxybutyrate (3OHB). Evidence indicates that the rise in BDNF can be attributed to the presence of 3OHB subsequent to physical activity [67].

In a recent study, Marosi and colleagues showcased the potential of 3OHB in activating the BDNF gene promoter, specifically within cerebral cortical neurons. This activation was accomplished through a unique signaling pathway that relies on the collaboration between the transcription factor nuclear kappa B (NF-κB) and the histone acetyltransferase p300 [121]. Furthermore, the autophagy-dependent nature of β hydroxybutyrate has been observed to facilitate the enhancing impact of physical activity on cognitive function and memorization [122].

### 2.15. Lactate

Lactate has a double duty, serving as both a fuel source and a messenger in the body. Astrocytes have the ability to absorb glucose or break down glycogen for energy production through glycolysis, resulting in the release of lactate into the space surrounding cells. Neurons can subsequently utilize this extracellular lactate as a vital source of energy, especially when there is increased synaptic activity. This connection between synaptic activity and energy transfer establishes a significant metabolic relationship.

Conversely, lactate plays a significant role as a messenger molecule, triggering a cascade of signaling pathways through specialized receptors. This stimulation prompts the activation of immediate early genes and facilitates the growth of blood vessels in the brain. Amidst moderate to intense physical activity, lactate production escalates both in muscles and the bloodstream, compelling the brain to actively consume lactate originating from skeletal muscles. As a result, this process boosts aerobic glycolysis, ultimately resulting in higher levels of lactate produced by the brain.

Exercise has a notable impact on the functionality and performance of transporters and enzymes responsible for the astrocyte-neuron lactate shuttle. This enhances the effectiveness of the process. Moreover, exercise triggers the activation of the lactate receptor known as hydroxycarboxylic acid receptor 1 (HCAR1), which subsequently influences brain plasticity [123].

The effect of exercise intensity on lactate in cognition impairment has been studied in some studies. One study found that high-intensity exercise [124] improved the acute cognitive response to a higher degree when compared to moderate-intensity exercise or control [125]. The cognitive improvements correlated with lactate release, providing a plausible molecular explanation for the cognitive enhancement [90]. Another study evaluated the effects of high-intensity functional training on general cognition in older adults with CI and found that a progressive high-intensity functional training program improves general cognition. The authors suggested lactate signaling as an important mediator [73,89].

### 2.16. PGC1-a

PGC1-a has been linked to alterations in neurocognitive function caused by exercise. Researches indicated that participating in exercise, either resistance or endurance, could increase the blood levels of PGC1-a, which can positively impact cognitive functions. One study suggested that the PGC1-α-dependent myokine, which stimulates the brown-fat-like growth of fatty tissue, may possess neuroprotective functions in AD [101,102,103].

In particular, it has been found that exercise-induced overexpression of PGC1-a can enhance the presence of kynurenine aminotransferases, which convert kynurenine to kynureniceacid (KYNA), an endogenous antagonist of N-methyl-d-aspartate and alpha 7-nicotinic acetylcholine receptor [124]. KYNA is also the antagonist of the α7 nAChR. KYNA level changes in brains may, therefore, affect the physiological functions related to α7-nicotinic acetylcholine neurotransmission [126]. Failure to maintain physiological concentrations of brain KYNA could be one of the causative factors leading to neuropathological conditions such as AD and CI [127].

### 2.17. Neurotransmitters and Neuromodulators

Physical activity leads to the release of two neurotransmitters, dopamine and 5-hydroxytriptamin (5-HT), which are essential for motor control and learning. These neurotransmitters have been observed to increase in various brain areas, such as the hippocampus, prefrontal cortex (PFC), stratum, and midbrain. Their presence is crucial in the regulation of adult neurogenesis within the hippocampus [77].

Neuromodulators are substances capable of modulating or regulating the activity of neurons. Neurotransmitters are directly involved in transmitting signals between individual neurons at synapses, while neuromodulators have a more widespread and modulatory influence on the overall activity and responsiveness of neural circuits, they play a pivotal role in cognitive function by influencing processes such as learning, memory, and attention. For instance, flavonoids, a category of dietary phytochemicals, have demonstrated positive effects on CNS [128]. They contribute to enhancing cognitive function by safeguarding neurons from stress-induced injury and suppressing neuroinflammation.

Researchers suggest that physical exercise could effectively impact neuromodulators, although study results are inconclusive and warrant further investigation [129].

One study showed that exercise could increase nerve growth factor (NGF) within the septohippocampal pathway, which is responsible for the enhancement of septo-hippocampal cholinergic structure and function. Recognizing the important link between exercise and NGF levels, it becomes evident that promoting physical activity can greatly aid in the improvement of failing septo-hippocampal functioning and potentially contribute to significant recovery in individuals with neurological disorders. However, the modulation of proNGF and proBDNF, two neurotrophins, is intricate and likely influenced by various pathways. These neurotrophin variations have previously been associated with depression, stress, and anxiety [130]. Castrillon et al. suggested that an up-regulation of neuromodulator activity (e.g., by physical activity), alongside increased brain size, is a crucial aspect of human brain evolution and can help in improving cognitive function [131].

### 2.18. Inflammation

Exercise suppresses chronic inflammation by inducing the release of IL-6 from skeletal muscles. IL-6 then triggers the production of IL-1 receptor antagonist (IL-1ra) and IL-10, which exert anti-inflammatory effects. IL-1ra blocks inflammation mediated by IL-1, while IL-10 inhibits the production of several pro-inflammatory cytokines, including IL-1α, IL-1β, IL-8, TNF, and macrophage inflammatory protein-α. The regulation of cellular metabolism in macrophages is another vital role played by IL-10, with the objective of minimizing inflammation. It achieves this by activating the mTOR inhibitor DDIT4, which inhibits glucose absorption and eliminates damaged mitochondria exhibiting low membrane potential and elevated reactive oxygen species [77].

The provided study and related research suggest that high-intensity aquatic-based exercise has cognitive benefits for older adults, particularly those with MCI [132,133]. The effects are believed to be mediated by alterations in pro- and anti-inflammatory protein markers.

Studies emphasize the potential of aquatic exercise to reduce the risk of late-life CI and dementia by influencing immune and inflammatory pathways. However, the need for larger studies to replicate these findings and further explore the role of activity-induced inflammatory changes on late-life brain health is also highlighted. The evidence presented underscores the potential of high-intensity aquatic exercise as an effective non-pharmacological intervention to improve cognitive function and mitigate cognitive decline in older adults, especially those with MCI [133].

### 2.19. Oxidative Stress

Physical activity promotes the growth of new mitochondria and enhances the functionality of antioxidant enzymes, benefiting not only the body but also the brain [134]. This dual effect contributes to the reduction of oxidative stress. Additionally, exercise aids in removing amyloid, a protein associated with neurodegenerative diseases ‚ by enhancing both the blood-brain barrier and the glymphatic system. Animal models of Alzheimer’s disease have shown that engaging in wheel running can prevent microglia activation and decrease oxidative stress and inflammation in the hippocampus and cortex. This, in turn, can lead to reduced amyloid deposition and enhanced spatial learning abilities, as observed in the Morris Water Maze and Barnes Maze etc. [135]. Running on a wheel has the added benefit of diminishing the amount of triggered microglia and deposits of amyloid while simultaneously augmenting the number of dendrites, dendritic spines, and postsynaptic density protein found in the cortex and hippocampus of regular elderly mice. This correlation improved the grasping of spatial concepts in the Morris Water Maze [136]. Older adults who engage in higher levels of physical activity, as measured by a pedometer, experience a decelerated cognitive decline related to amyloid accumulation and a reduction in the volume of gray matter, which is where neuron cell bodies reside within the brain [137].

Recent findings collectively suggest that exercise intensity can influence oxidative stress [138], and higher intensities of aerobic exercise would be the best in reducing oxidative stress [90,99].

### 2.20. Beneficial Effect of Stem Cells 

Athletes’ plasma has been found to contain increased levels of various substances‚ such as vascular endothelial growth factor (VEGF), IL-6, nitric oxide (NO), granulocyte colony-stimulating factor (G-CSF), granulocyte macrophage colony-stimulating factor (GM-CSF), stromal cell-derived factor-1α (SDF-1α), and hypoxia-inducible factor-1α (HIF-1α). This elevation in levels is known to stimulate the mobilization of endothelial progenitor cells from the bone marrow [139,140,141,142,143,144,145,146,147,148,149,150]. Enhancement of endothelial function, restoration of damaged endothelium through new cell production, and promotion of neovascularization lead to the restoration of BBB integrity, culminating in reduced levels of neuroinflammation [139,140,141,142,143,144,145,146,147,148,149,150].

Moreover, SDF-1α facilitates the attraction of neural stem/progenitor cells (NSCs) towards the site of brain injury by engaging with its receptor, chemokine receptor type 4 (CXCR4). CXCR4 is predominantly found on neural stem/progenitor cells, and cells that express CXCR4 migrate in response to the concentration gradient of SDF-1α [151,152]. NSCs are responsible for neurogenesis and brain plasticity that could replace dead neurons [153]. Consequently, the process of triggering the enlistment and relocation of NSCs to the location of the harm ultimately results in the production of fresh neural cells [154,155,156].

Monocarboxylate transporters (MCTs) play a crucial role in regulating lactate levels in the extracellular region of the brain. Hence, when MCT1 is lacking in brain endothelial cells (ECs), it results in elevated lactate levels. These increased lactate levels have a dual effect—causing heightened depletion of the NSC (neural stem cell) pool and a decline in the production of fully developed newborn neurons within the hippocampus [157]. The activity of runners’ plasma has the potential to activate MCT1 expression in the endothelial cells of the brain. This, in turn, can regulate the growth and development of neural stem cells, ultimately leading to the restoration of cognitive function [123,157].

Mesenchymal stem cells (MSCs) are a type of stem cell that has the ability to differentiate into neurons. The brain-damaged regions receive a boost in the number of progenitor and proliferating cells when MSCs are stimulated to migrate using SDF-1 and G-CSF cytokines [158]. Furthermore, the potential of MSCs to enhance cognitive function arises from their ability to exhibit anti-inflammatory properties, modulate the immune system, and prevent cell death [159]. In addition, the impact of MSCs on the bone marrow extends further, stimulating the growth of endothelial progenitor cells and circulating endothelial cells (ECs). This process ultimately leads to the remodeling of the BBB in individuals with AD [160]. Injecting runner plasma has the potential to enhance both the proliferation rate and total quantity of MSCs in bone marrow [161].

## 3. Exercise Prescription Guidelines

Type of Exercise: Both aerobic exercise (AE) and resistance exercise (RE) have been shown to improve cognitive function in older adults, including those with MCI. AE includes activities like walking, swimming, and cycling, while RE involves strength training with bodyweight or weights. Research indicates that high-intensity exercise, such as high-intensity functional training (HIFT) and high-intensity interval training (HIIT), may have potential effects on cognitive performance in individuals with cognitive impairment. For instance, a systematic review of randomized controlled trials found that a progressive HIFT program improved general cognition in older adults with CI, while another study compared the effects of HIIT and moderate continuous exercise training on cognitive performance in persons with multiple sclerosis (pwMS), suggesting potential benefits of high-intensity exercise on cognitive performance in this population.

Personalization: Exercise prescription should be personalized based on deficits in specific cognitive domains. For individuals who may have mobility issues, RE can be recommended as an alternative to AE.

Duration and Intensity: while specific durations are not mentioned in the search results, exercise interventions are suggested to be longer than four weeks, and the intensity should be tailored to the individual’s fitness level and cognitive abilities.

Supervision and Adherence: Effective supervision and monitoring of exercise training are important to ensure safety and efficacy. Encouraging adherence to the exercise program is crucial for maintaining cognitive function and preventing cognitive decline.

In summary, the guidelines for exercise prescription to promote cognitive function emphasize the personalization of exercise based on individual cognitive deficits, the inclusion of both aerobic and resistance exercises, and the importance of supervision and adherence to the exercise program [73,89,162,163,164].

## 4. Conclusions

Exerkines confer a myriad of benefits that contribute significantly to enhanced cognition. This encompasses the release of growth factors, neurotransmitters, and cytokines, along with the elevation of specific metabolites such as lactate and ketone bodies. Moreover, exerkines are intricately linked to a reduction in inflammation and oxidative stress, thereby mediating the observed cognitive improvements. Furthermore, the potential of exerkines extends to the facilitation of increased stem cell mobilization. This, in turn, can exert anti-inflammatory, immunomodulatory, and antiapoptotic effects, ultimately promoting cognitive function.

This comprehensive understanding of the intricate mechanisms involved not only underscores the pivotal role of exerkines but also lays the groundwork for developing targeted interventions to harness the full cognitive benefits of exercise.

## Figures and Tables

**Table 1:** The effects of exerkines on CI.

Exerkines	Effects on Cognitive Impairments
Interleukin-6 (IL-6)	Stimulates the growth of new neurons in the hippocampus
Cathepsin B (CTSB)	Neuroprotective effects, especially in hippocampal neurogenesis and neuronal migration
Brain-derived neurotrophic factor (BDNF)	Neuronal differentiation and survival, synaptic integrity, brain plasticity, memory, and cognitive flexibility
Glial-derived neurotrophic factor (GDNF)	Gliogenesis
Irisin	Cell proliferation and BDNF expression in brain tissue, contributing to the generation of new neurons, protection to neurons against the toxic effects of Aβ
Fractalkine (FNDC5/irisin)	Activates (cAMP/PKA/CREB) in brain, promotion of hippocampal neurogenesis, improve cognitive function
Apelin	Neuroprotective
Clusterin	Facilitating Aβ clearance across the BBB
C-X3-C Motif Chemokine Ligand 1 (CX3CL1)	Implicated in memory-associated synaptic plasticity in the rat hippocampus
Fibroblast growth factor 2 (FGF2)	Promote the growth of new neurons and blood vessels in the brain
Fibroblast growth factor 21 (FGF21)	Hindered the formation of amyloid plaques, neurofibrillary tangles, and overall neurodegeneration
Insulin-like Growth Factor-1 (IGF-1)	Neurogenesis
Leukemia-inhibitory factor (LIF)	Growth and development of neurons
Vascular endothelial growth factor (VEGF)	Neurogenesis in the dentate gyrus of the hippocampus, angiogenesis, improved results in a short-term memory assessment
3-Hydroxybutyrate (3OHB)	Rise in BDNF can be attributed to the presence of 3OHB subsequent to physical activity, enhancing cognitive function and memorization by the effect of the autophagy-dependent nature of β-hydroxybutyrate
Lactate	Fuel source of brain, impact on the functionality and performance of transporters and enzymes responsible for the astrocyte-neuron lactate shuttle, brain plasticity
Peroxisome proliferator-activated receptor-gamma coactivator (PGC)-1α	Neuroprotective
Anti -inflammatory cytokines	IL-6 triggers the production of IL-1 receptor antagonist (IL-1ra) and IL-10, which exert anti-inflammatory effects. IL-1ra blocks inflammation mediated by IL-1, while IL-10 inhibits the production of several pro-inflammatory cytokines, including IL-1α, IL-1β, IL-8, TNF, and macrophage inflammatory protein-α. The regulation of cellular metabolism in macrophages is another vital role played by IL-10

## Data Availability

The original contributions presented in the study are included in the article further inquiries can be directed to the corresponding authors.
